# 3D-printed model-guided endoscopic evacuation for basal ganglia hemorrhage

**DOI:** 10.1038/s41598-020-62232-3

**Published:** 2020-03-23

**Authors:** Jun Zhang, Hongyu Cheng, Sitong Zhou, Lijin Huang, Jianguang Lv, Peng Wang, Jiajing Chen, Tongxin Jin, Guiyong Zheng, Haoxiang Ye, Xuejie Wang, Bing Meng, Dan Lu, Yuqian Li

**Affiliations:** 1 Department of Neurosurgery, Dalang Hospital, Dongguan, China; 2 Department of Ultrasound Diagnostics, Tangdu Hospital, Air Force Medical University, Xi’an, China; 3 The Medical College of Zhengzhou University, Zhengzhou, China; 4 Department of Neurosurgery, The Third Affiliated Hospital of Southern Medical University, Guangzhou, China; 5 Intensive Care Unit, Dalang Hospital, Dongguan, China; 6 Department of Anesthesiology, Dalang Hospital, Dongguan, China; 7 Department of Radiology, Dalang Hospital, Dongguan, China; 8 Department of Neurosurgery, Shipai Hospital, Dongguan, China; 9 Department of Neurosurgery, Xi’an International Medical Center, Xi’an, China; 10 Department of Neurosurgery, Tangdu Hospital, Air Force Medical University, Xi’an, China

**Keywords:** Stroke, Stroke

## Abstract

The purpose of this study was to investigate the effectiveness and practicality of 3D-printed model-guided endoscopic surgery for the treatment of basal ganglia hemorrhage. The authors retrospectively analyzed the data of all patients who underwent endoscopic evacuation of basal ganglia hemorrhage in the Department of Neurosurgery at Dalang Hospital and Shipai Hospital between December 2017 and February 2019. Twelve patients, in whom the 3D-printed model guidance was used for endoscopic evacuation, were included in this investigation. Using 3D reconstructed technology, we designed the appropriate surgical approach. Then, an individualized facial model with the guide orifice was printed by a 3D printer. Further, the 3D-printed model was employed to guide the insertion of the endoscope sheath. As a result, the average evacuation rate was 97.2% (range 90.1–100.0%). The GCS and mRS score were improved in each patient from admission to discharge examination. All patients had a good prognosis based on their functional independence measure (FIM) scores at the 6-month follow-up. The 3D-printed model-guided endoscopic evacuation was effective and safe for basal ganglia hemorrhage. This technique deserves further investigation to determine its role in intracerebral hemorrhage management.

## Introduction

Hypertensive intracerebral hemorrhage (HICH) is a very common neurosurgical disease disorder with a poor prognosis and outcome. The hematoma is generally located in the brain parenchyma such as the basal ganglia^1–3^. Recently, endoscopic surgery for the hematoma evacuation has gradually replaced craniotomy to minimize brain injury^4,5^. Endoscopic techniques is a new method of minimally invasive operation that have the advantages of less trauma, fewer complications, and better prognosis. Compared with traditional craniotomy, the accurate localization of the hematoma is more important in endoscopic surgeries. Precise insertion of the puncturing needle or sheath is the crucial factor of the accomplishment of operation^6,7^. Hence, we need a low-cost and easy-to-follow technique to localize the deep-seated hematoma.

3D printing technology is based on the original digital imaging and communications in medicine (DICOM) data of CT and 3D software modeling. On the basis of this technology, the shape and position of hematomas in patients are analyzed, and the surgical approach is designed to avoid important positions, such as those of the venous sinus, the functional area, and the forehead sinus^8^. According to the facial features of the patients, an individualized facial model with the guide orifice is designed and printed by a 3D printer. In the hematoma puncture process, the puncture angle and the puncture position can be fixed through the guide orifice, which reduces the tissue injury, caused by the inaccurate localization and repeated puncture. This method for the treatment of HICH by neuroendoscopy is personalized and more accurate than the conventionally used ones.

In the present study, we retrospectively analyzed the data of 12 HICH patients using low-cost and easy-to-follow 3D model guidance for evacuation of basal ganglia hematomas.

## Results

The patients’ clinical characteristics and surgical details are presented in Tables 1 and 2. Of the 12 patients included, 6 were female and 6 were male, with an age ranging from 41 to 67 years. All the patients had hypertension history. No brain hernia and occlusive hydrocephalus was observed before surgery. Three of the patients had intraventricular hemorrhage (IVH) with a Graeb Scale score of 1–2. No patients underwent external ventricular drainage before the endoscopic evacuation. There was no rebleeding, and no patients were subjected to revision surgery after the endoscopic evacuation. The mean operating time was 102.4 min (range 81.0–133.0 min). The mean intraoperative blood loss volume was 50.1 mL (range 20–100 mL).

**Table 1:** Details of HICH patients at admission.

Case NO.	Patient characteristics					CT findings				
	Age (years)/Sex	BMI	SBP at admission (mmHg)	Aspirin history (yes/no)	Diabetes history (yes/no)	Location	HV excluding IVH (mL)	IVH (yes/no)	Graeb Scale (score)	Hydrocephalus (yes/no)
1	38/M	23.4	162	No	No	Right	46.6	No	0	No
2	42/F	23.0	147	No	No	Right	33.4	No	0	No
3	46/F	20.1	151	No	Yes	Left	39.3	No	0	No
4	49/M	21.5	166	No	No	Left	57.5	No	0	No
5	52/F	23.1	159	No	No	Right	51.6	No	0	No
6	52/F	22.4	172	No	No	Right	66.3	Yes	1	No
7	53/M	25.0	153	No	No	Left	40.8	No	0	No
8	55/F	23.8	169	No	No	Left	49.6	No	0	No
9	55/M	24.9	185	No	Yes	Right	72.5	Yes	2	No
10	58/F	25.2	172	No	No	Right	55.6	No	0	No
11	63/M	26.7	155	No	No	Left	47.7	No	0	No
12	67/M	22.4	160	No	No	Right	58.4	No	0	No

HICH: hypertensive intracerebral hemorrhage; HV: hematoma volume; GCS: Glasgow coma scale; SBP: systolic blood pressure; IVH: intraventricular hemorrhage.

**Table 2:** Surgical details.

Case NO.	OT (min)	IBL (mL)	EVD (yes/no)	RAS (yes/no)	RS (yes/no)
1	109	50	No	No	No
2	96	42	No	No	No
3	102	20	No	No	No
4	127	40	No	No	No
5	81	30	No	No	No
6	93	40	No	No	No
7	88	60	No	No	No
8	85	60	No	No	No
9	133	100	No	No	No
10	99	30	No	No	No
11	112	60	No	No	No
12	104	70	No	No	No

OT: operative time; IBL: intraoperative blood loss; EVD: external ventricular drainage; RAS: rebleeding after surgery; RS: revision surgery.

The data of the clinical efficacy of the facial model-guided endoscopic evacuation are presented in Table 3. The mean hematoma volumes before and after operation were 50.6 mL (range 33.4–72.5 mL) and 1.4 mL (range 0.0–3.9 mL), respectively, with an average evacuation rate of 97.2% (range 90.1–100.0%). In each patient, the GCS and mRS scores improved on discharge compared with those on admission. The median admission GCS and mRS scores were 9.5 and 5, correspondingly. The median discharge GCS and mRS scores were 12 and 3, respectively. Six months after the operation, the median FIM score was 122 (range 109–126). All patients had a good prognosis based on FIM scores. Of the 12 patients, 4 were evaluated as complete independence (126 points), 8 were evaluated as independence despite slight symptoms (108–125 points).

**Table 3:** Summary of the imaging and clinical results.

Case NO.	HV(mL)		ER (%)	GCS Score		mRS Score		FIM at six months (score)
	Preop	Postop		Admission	Discharge	Admission	Discharge	
1	46.6	0.0	100.0	11	14	4	1	126
2	33.4	0.4	98.7	11	14	4	2	126
3	39.3	3.9	90.1	9	12	5	3	123
4	57.5	0.8	98.6	10	12	5	3	120
5	51.6	0.5	99.1	11	13	4	2	126
6	66.3	1.8	97.3	8	11	5	4	112
7	40.8	1.8	95.6	10	14	4	2	126
8	49.6	2.2	95.5	9	12	5	4	118
9	72.5	2.6	96.4	7	10	5	4	109
10	55.6	0.9	98.3	9	12	5	3	121
11	47.7	0.3	99.3	11	13	5	3	123
12	58.4	1.8	96.9	9	11	5	4	113

HV: hematoma volume; ER: evacuation rate; GCS: Glasgow coma scale; mRS: modified ranking scale; FIM: functional independence measure.

The average length of stay in the hospital was eight days (Table 4). No severe systemic complication occurred (Table 4). Major complications had developed in three patients (25.0%). These included pulmonary infection in two patients (cases 9 and 12), digestive tract ulcer in 1 patient (case 6). These complications were cured by corresponding treatment and nursing.

**Table 4:** Surgery results.

	Patient data
Hospital LOS (days)	8 ± 2.5
Death	0 patient
**Complications**	
Pulmonary infection	2 patients
Digestive tract ulcer	1 patient
Hypoproteinemia	0 patients
Epilepsy	0 patients
Intracranial infection	0 patients

LOS: length of stay. Data are expressed as mean ± SD or number of patients.

### Illustrative cases

**Figure 1:**
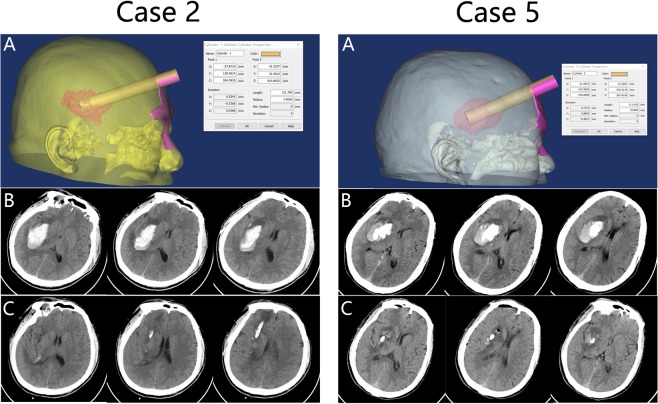
(**A**) 3D reconstruction and computer-assisted planning were used to determine the trajectory of the puncturing needle. Preoperative (**B**) and postoperative (**C**) CT scans for case 2 and 5 in this study.

The preoperative and postoperative CT scans of Cases 2 and 5 in this study can be seen in Fig. 1. The preoperative CT scans confirmed the presence of hematoma in the right basal ganglia of these two patients (Fig. 1B**)**. Before the operation, we adopted 3D reconstruction and computer-assisted planning for identification of burr hole location, puncture trajectory, and depth of sheath insertion (Fig. 1A**)**. Based on the CT data, 3D printed facial model was created. Then, 3D model-guided endoscopic evacuation of the hematoma was performed. The postoperative CT scan confirmed the almost complete evacuation of the hematoma (Fig. 1C**)**.

## Discussion

Here, we report how 3D imaging and printing technology can be used to improve the clinical outcomes of endoscopic evacuation of ICH. In our series of 12 patients, the mean reduction of hematoma volume was 97.2%. The patients showed a significant improvement in GCS and mRS scores at discharge, and FIM score at the six-month follow-up. These results demonstrated that 3D facial model-guided endoscopic evacuation of ICH is effective and safe.

Compared with craniotomy, endoscopic evacuation of ICH has several advantages, such as minimal invasive procedure, fewer complications, higher magnification, shorter operation period, and better prognosis^5,9,10^. It has a broad prospect in the surgical treatment of HICH^11^. In the last two decades, the endoscopic evacuation rate of intracerebral hematomas has been rather low, because the narrow hematoma cavity can obstruct the endoscopic view^6,12^. The advent of the endoscopic sheath improved the visualization of the surgical field and the removal of the hematoma^12,13^. When the surgeon place the sheath into the core of the hematoma, the collapsing hematoma cavity may push the surrounding clot into the tip of the sheath during the evacuation process^12^. Hence, the accurate placement of the sheath into the the core of the hematoma is exceedingly important for this endoscopic surgery technique. Currently, accurate localization is generally based on image guidance or real-time ultrasound guidance^5,9,10,14^. CT-image-guided evacuation of intracerebral hemorrhage was widely used^15,16^. Many medical centers routinely adopted framed or frameless navigation systems. However, such navigation systems are rather expensive and unavailable in many developing regions^17^. In addition, the navigation is rarely used in emergency circumstance due to the time-consuming preparation. Ultrasound guidance for the sheath insertion is a real-time navigation, but it requires extra space for placing the ultrasound probe, which may cause additional injury to the patient.

The rapid development of 3D printing technology provides new methods for the navigation of the neuroendoscopic technique. This is the first report of 3D-printed model-guided endoscopic evacuation of ICH-related hematomas. In this study, we obtained accurate hematoma localization in all 12 cases included. Under the aid of the 3D reconstructed CT scan techniques, we can determine an accurate trajectory to the core of hematoma with the minimized brain trauma during manipulation of the endoscope. Based on the preoperative thin-slice CT scan and computer-assisted design, the designed file can be exported to 3D printing equipment in fixed format so as to allow for printing of an individualized facial model with an accurate puncture tunnel for the endoscopic operation. This model can reduce the swing of the puncture device in the process of puncture, and the fixed puncture angle can avoid frontal sinus and related important blood vessels, and prevent massive bleeding and unnecessary injury caused by the wrong direction of the puncture. The whole operation process is more convenient and accurate, with reduced operation time, and avoided possibility of repeated puncture. Using the 3D model guidance technology combined with neuroendoscopic technique to remove hematoma is cheap, simple, and fast. Compared with the traditional navigation systems, it offers much more remarkable social benefits and is more suitable for popularization and application in local hospitals and even nationwide.

In the present article, we have described the 3D model guidance as an alternative to image guidance for endoscopic evacuation of ICH. Imaging results showed a significant reduction in hematoma volume and revealed no episodes of recurrent postoperative bleeding. The clinical outcomes exhibited a trend toward improvement of our patients’ condition. However, the procedure needs to be tested in a greater number of patients to obtain statistically significant data. Nonetheless, certain limitations and concerns about this 3D model-guided endoscopic technique for ICH are to be acknowledged. First, this retrospective review included only a small case series of basal ganglia hemorrhage, with no control treatment by other surgical techniques or a comparison with conservative management. A large prospective study is thus highly desirable to confirm our findings. Second, this approach is not suitable for patients with cerebral hernia, where decompressive craniectomy is recommended.

## Conclusions

A high removal rate can be safely achieved in endoscopic surgery for basal ganglia hemorrhage by using the 3D facial model guidance. This novel approach is suitable for treatment of basal ganglia hemorrhages, which commonly have an elongated ovoid shape. The trajectory afforded by computer-assisted design has the advantages of minimal trauma and good effect.

## Methods

### Ethical approval and informed consent

This study was approved by the Medical Ethical Committees of Dalang Hospital and Shipai Hospital. The experimental protocol was established, according to the ethical guidelines of the Helsinki Declaration. Written informed consent was obtained from all study participants. All methods were performed in accordance with the relevant guidelines and regulations. This study dose not contain any identifying information that could lead to identification of the participant.

### Patients

From December 2017 to February 2019, 25 consecutive patients with HICH were were prospectively enrolled into the study. After obtaining written and informed consent, we adopted computer-assisted planning and 3D model guidance for endoscopic hematoma evacuation in 12 patients. All the 12 patients presented with basal ganglia hemorrhage. These patients undergone endoscopic hematoma evacuation through the frontal bur-hole approach. This approach was suitable for elongated or ovoid-shaped basal ganglia hematoma. The perioperative care of the patients was decided by individual surgeons and anesthetists, based on relevant guidelines and regulations.

All the patients were diagnosed by craniocerebral CT and contrast-enhanced CT. Both examine were used for ruling out brain arteriovenous malformation, and for 3D reconstruction. The hematoma volumes (HV) were estimated by 3D printing software Mimics 16.0. Each patient was initially admitted to the neurosurgical ICU and given preoperative preparation. Systolic blood pressure (SBP) was controlled and maintained between 110 and 140 mmHg by urapidil infusion.

The following patient inclusion criteria were used in this study: (1) Hematoma was from the basal ganglia, with an elongated ovoid shape; (2) Cerebral hemorrhage volume > 30 mL; (3) No serious visceral diseases or clotting disorders; (4) No cerebral hernia; (5) Stable vital signs; (6) GCS scores ≥ 5

The exclusion criteria were as follows: (1) GCS scores <5; (2) Hemorrhage in the cerebellum, subcortex, thalamus and brain stem; (3) Serious visceral disease or clotting disorders; (4) Cerebral aneurysms, vascular malformation, trauma, tumor, cavernous malformations, and hemorrhagic conversion of cerebral infarction; (5) Ongoing hematoma expansion; (6) Lost follow-up.

### 3D printed facial model

**Figure 2:**
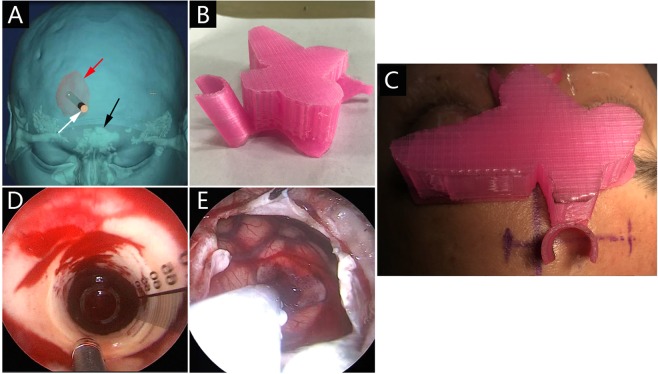
The surgical procedure of endoscopic evacuation using 3D facial model guidance. (**A**) 3D reconstruction was used to plan the trajectory of the endoscopic sheath (white arrow). Frontal sinus (black arrow), hematoma (red arrow). (**B**) The 3D printed facial model with extracranial guide orifice. (**C**) The 3D facial model was fixed onto the patient’s face. (**D**) Endoscopic view of suction catheter approaching hematoma bed through the sheath. (**E**) After eliminating the hematoma, the sheath was removed, the brain tissue around the channel retracted.

At admission, all patients underwent thin-slice CT scans of the skull. The DICOM files of the CT image were imported into the 3D printing software Mimics 16.0 for 3D reconstruction. The 3D reconstructed images of the hematoma and the patient’s scalp were superimposed on the digital computer. Through computer-assisted design, this system was used to identify the appropriate entry point, the trajectory to enter the hematoma, and the depth of endoscope insertion. The average time for scanning and reconstruction was 15 min at most. On the 3D reconstructed CT image, the hematoma center was superimposed on the scalp by the anterioposterior (AP) axis, and then the frontal entry point was determined. The frontal approach was applied for each patient in this series, in which the entry point did not involve the frontal sinus (Fig. 2A). We used the nasion as the reference point for the determination of the frontal entry point. The distance between the nasion and the frontal entry point was measured on the 3D reconstructed CT images. The distance between the frontal entry point and the hematoma center was calculated on the 3D reconstructed CT image. Then, the 3D image file of head was employed to construct the 3D facial model with extracranial guide orifice, which was then printed using a 3D printer (CASET 250MC, China) (Fig. 2B). Polylactic acid (PLA) was utilized as the raw material, and the printing technology in this study was called Fused Filament Fabrication (FFF). The 3D printed facial model was further customized for each patient. The inner surface of the facial model was trimmed until an exact fit to the patient’s face was reached, and the model would not rotate or displace after fixation. This model was then sterilized, and was prepared for the surgery.

### Endoscopic procedure

After general anesthesia, the patient was placed in a supine position. The intraoperative neuronavigation was not used in any case, and was replaced by the 3D model. The 3D facial model was fixed onto the patient’s face (Fig. 2C). Based on the data calculated on 3D reconstructed CT image, the frontal entry point was under the extracranial guide orifice of 3D facial model. The burr-hole position and the direction desired for the puncturing needle were determined by guide orifice of the model. A 5–6-centimeter incision was made, consistent with the planned endoscopic trajectory. A 3 cm burr hole was created using a Surgical Power Device (DK-N-MS, XISHAN, China). Using the facial model guidance, a puncturing needle was inserted into the predetermined center of the hematoma through the guide orifice of the model. Next, a 10-ml syringe was applied for suction to reduce the intracranial pressure and to further confirm whether the tip of the needle was in the hematoma. The we introduced a transparent sheath to an expected depth along the puncturing needle. Through the transparent sheath, a neuroendoscope (diameter 4 mm, 0°, Youshi Medical instrument) was inserted. When the endoscope was put in place, we had clear visualization of the brain parenchyma and the border of the hematoma through the transparent sheath (Fig. 2D). The technique of the endoscopic surgery was performed as previously reported by Dye *et al*.^14^. The hematoma cavity was irrigated with saline. If there was active bleeding from the surface of hematoma cavity, A insulated cannula for suction and coagulation (TH0852-007, THINK, Germany) was used as needed, and this monopolar can worked through the cannula tip while the bleeding was controlled with the suction. After sufficient hematoma evacuation and decompression, the transparent sheath was removed, and the brain tissue around the channel was retracted (Fig. 2E). A ventriculostomy catheter was left in the hematoma cavity for 48 hours to drain the early recurrent bleeding.

### Imaging and clinical evaluation

Postoperative CT is performed within eight hours after surgery, to determine the effect of surgery. The hematoma evacuation rate (ER) was defined as 100 − (postoperative volume)/(preoperative volume) × 100%. We use the Glasgow Coma Scale (GCS) and modified Rankin Scale (mRS) to assess the neurological status from admission to discharge. Postoperative follow-up was performed six months postoperatively, and the Functional Independence Measure (FIM) scale was employed for clinical efficacy evaluation.
